# Molecular Mimicry Mapping in *Streptococcus pneumoniae*: Cues for Autoimmune Disorders and Implications for Immune Defense Activation

**DOI:** 10.3390/pathogens12070857

**Published:** 2023-06-21

**Authors:** Mutaib M. Mashraqi, Ahmad Alzamami, Norah A. Alturki, Saleh Alshamrani, Mousa M. Alshahrani, Hassan H. Almasoudi, Zarrin Basharat

**Affiliations:** 1 Department of Clinical Laboratory Sciences, College of Applied Medical Sciences, Najran University, Najran 61441, Saudi Arabia; mmmashraqi@nu.edu.sa (M.M.M.); saalshamrani@nu.edu.sa (S.A.); mmalshahrany@nu.edu.sa (M.M.A.); 2 Clinical Laboratory Science Department, College of Applied Medical Science, Shaqra University, AlQuwayiyah 11961, Saudi Arabia; aalzamami@su.edu.sa; 3 Clinical Laboratory Science Department, College of Applied Medical Science, King Saud University, Riyadh 11433, Saudi Arabia; noalturki@ksu.edu.sa; 4 Alpha Genomics (Private) Limited, Islamabad 45710, Pakistan

**Keywords:** *Streptococcus pneumoniae*, molecular mimicry, bioinformatics, HLA, autoimmune disorders

## Abstract

*Streptococcus pneumoniae* contributes to a range of infections, including meningitis, pneumonia, otitis media, and sepsis. Infections by this bacterium have been associated with the phenomenon of molecular mimicry, which, in turn, may contribute to the induction of autoimmunity. In this study, we utilized a bioinformatics approach to investigate the potential for *S. pneumoniae* to incite autoimmunity via molecular mimicry. We identified 13 *S. pneumoniae* proteins that have significant sequence similarity to human proteins, with 11 of them linked to autoimmune disorders such as psoriasis, rheumatoid arthritis, and diabetes. Using in silico tools, we predicted the sequence as well as the structural homology among these proteins. Database mining was conducted to establish links between these proteins and autoimmune disorders. The antigenic, non-allergenic, and immunogenic sequence mimics were employed to design and validate an immune response via vaccine construct design. Mimic-based vaccine construct can prove effective for immunization against the *S. pneumoniae* infections. Immune response simulation and binding affinity was assessed through the docking of construct C8 to human leukocyte antigen (HLA) molecules and TLR4 receptor, with promising results. Additionally, these mimics were mapped as conserved regions on their respective proteins, suggesting their functional importance in *S. pneumoniae* pathogenesis. This study highlights the potential for *S. pneumoniae* to trigger autoimmunity via molecular mimicry and the possibility of vaccine design using these mimics for triggering defense response.

## 1. Introduction

*Streptococcus pneumoniae* is a Gram-positive bacterium with different serotypes distinguished by variations in the chemical composition of the capsule [1]. It causes invasive diseases, such as pneumonia, meningitis, and sepsis [2], as well as non-invasive pneumococcal disease, such as otitis media or sinusitis [3]. The bacterium inhabits the nasopharynx and can be spread through respiratory droplets or by direct contact with infected individuals [4]. It commonly infects young children and the elderly, causing morbidity, and even mortality [5]. It has several virulence factors, including pneumolysin, which can cause damage to host cells and contribute to inflammation and tissue damage [6]. There are approximately 100 known serotypes of *S. pneumoniae* [7], and vaccines have been developed to target the most prevalent ones [8]. Vaccine use has been associated with a decline in the occurrence of invasive pneumococcal disease among both pediatric and adult populations [9]. The treatment of pneumococcal infections typically involves antibiotics [10], although the advent of antibiotic-resistant strains is a growing concern [11].

Molecular mimicry is a crucial phenomenon in the development of autoimmune diseases, and understanding the involved molecular interactions can help in the development of targeted therapies to treat or prevent these diseases [12]. It is a phenomenon by which the immune system mistakenly identifies a host’s own tissues or molecules as a foreign entity and mounts an attack against them, leading to autoimmune disease. Certain pathogens have molecules on their surfaces that resemble host molecules, causing the immune system to mistakenly attack the host in addition to the pathogen [13]. A typical example of this occurs in rheumatic fever, an infection caused by *Streptococcus pyogenes.* It triggers an autoimmune response against host tissues, leading to inflammation of the heart, joints, and other tissues [14]. This occurs due to the streptococcal M protein, similar to the proteins found in human heart valve tissue, causing the immune system to attack the heart valves in addition to the streptococcal bacteria. Likewise, in multiple sclerosis, the immune system targets and damages the myelin surrounding the nerve cells of the brain and spinal cord, along with oligodendrocytes, leading to lesions in white and gray matter [15,16].

Bioinformatic-based approaches can be used to study molecular mimicry by analyzing large datasets of protein sequences and structures to identify similarities between host and pathogen molecules [17] via sequence and structural alignment [18]. Structural alignment can identify specific folds or structures that are conserved between the two proteins and may be responsible for binding specific receptors and initiating the cross-reactivity observed in molecular mimicry. Furthermore, bioinformatics allows for the prediction of immunogenic epitopes against molecular-mimicry-inciting proteins and the identification of sequence mimics. The immune system can identify these epitopes and instigate a defense response. Sequence conservation analysis of these mimics can assist in optimizing the vaccine design to ensure broad coverage against different strains or variants of the pathogen. 

Computational vaccine design can play a pivotal role in this endeavor, as utilizing bioinformatics tools and algorithms to predict and evaluate the immunogenicity and efficacy of vaccine constructs can give clues for the immunogenic response trigger [19]. Through the simulation approach, interactions between vaccine candidates and the immune system can be replicated, which can help infer the binding affinity of antigens to the immune receptors, leading to the subsequent activation of an immune response. Hence, this strategy enables the identification of optimal vaccine formulations that elicit strong and specific immune reactions while minimizing the potential side effects or unwanted immune cross-reactivity. We explore the utility of bioinformatic techniques in identifying relevant interactions between *S. pneumoniae* and human immune system in this research paper. By comprehending the precise molecular interactions involved, we can advance the development of focused therapeutic strategies to treat and prevent these complex autoimmune disorders. Additionally, sequence mimics can be used to design robust vaccine constructs against *S. pneumoniae.*

## 2. Material and Methods

The complete proteome of human and *S. pneumoniae* strain Hu17 (GenBank accession: CP020549.1) was retrieved from Uniprot (uniprot.org; accessed 1 May 2023) and the NCBI database (ncbi.nlm.nih.gov; accessed 1 May 2023), respectively. *S. pneumoniae* proteins were screened against the human proteome, using locally installed BLAST [20]. Homologous proteins, depicting a threshold of >50% identity and ≥100 bit-score, were retained for analysis.

### 2.1. Mimicry Prediction

Proteins were aligned to find regions of similarity (length ≥ 10 amino acids) that could be involved in mimicry. To appraise the peptide structural similarity, the root mean square deviation (RMSD) was employed as a scoring metric [18]. Three-dimensional structures of these proteins were obtained from AlphaFold, a database consisting of state-of-the-art predicted protein structures [21]. The obtained structures were then superposed using iPBA [22] and TM-align algorithms [23]. iPBA and TM-align are widely recognized tools in the field of structural biology for accurately comparing protein structures and determining their alignment on the basis of various structural features. These algorithms enhance the analysis by providing robust and reliable methods for measuring structural similarity and aligning proteins for further investigations. 

### 2.2. Pathway Enrichment

pathDIP [24], PHAROS (https://pharos.nih.gov/targets/; accessed on 4 May 2023) [25], and the literature were scanned for homologous protein pathways involved in autoimmune disorders or infections. Parameters for PathDiP included the following—Databases: BioCarta, ACSN2, SMPDB, HumanCyc, SIGNOR2.0, EHMN, IPAVS, INOH, NetPath, KEGG, OntoCancro, PharmGKB, Panther_Pathway, RB-Pathways, PID, REACTOME, systems-biology.org, stke, SignaLink2.0, Spike, WikiPathways, and UniProt_Pathways; Minimum confidence level: 0.99; Data type: extended pathway associations; and Protein interaction set: Experimentally detected and computationally predicted PPIs (full IID). These databases serve as a comprehensive references for signaling cascades in various species, encompassing key pathways sourced from major curated pathway databases. The association is based on both computational predictions as well as experimentally confirmed interactions. It also accounts for pathways predicted through orthology mapping and inference of physical interactions of the proteins. 

### 2.3. Mimic-Based Vaccine Construct Design

A successful mimic holds the potential to facilitate the development of a vaccine against a pathogen. Vaccines operate by triggering the immune system to generate antibodies targeting the specific protein of interest and defend against infections. By leveraging the mimicry mechanism, a vaccine can effectively elicit an immune response that safeguards the host from potential infections [25,26]. Hence, a reverse vaccinology approach was employed to design a vaccine construct against *S. pneumoniae*. For this purpose, the antigenicity of the mimics was determined using VaxiJen server [27], and immunogenicity was predicted using IEDB server [28]. Interleukin induction was also predicted using IL-4, IL-6, and IL-13 Pred server using default parameters (https://webs.iiitd.edu.in/raghava/; accessed 13 May 2023).

Peptide mimics were then checked for toxicity and allergenicity. Top immunogenic hits were selected for evolutionary conservation analysis using the ConSurf tool [29], with an iteration E-value: 0.0001. ConSurf utilizes HMMER algorithm and analyzes evolutionary patterns on the basis of conservation scores. Protein sequences were clustered using CD-HIT with a 95% cutoff, resulting in a maximum of 150 homologs. To avoid redundancy, a maximal overlap of 10% was allowed, selecting the highest-scoring homolog. Homologs covering at least 60% of the query sequence were considered. Multiple sequence alignment was performed using CLUSTALW [30] with the amino acid substitution model:WAG. The conservation scores were assessed through the Bayesian method, and then epitopes were predicted for these peptides using reference HLA set of alleles for MHC-I and II and using the IEDB server (http://tools.iedb.org/; accessed on 10 May 2023). Inferred epitopes were used for designing the vaccine construct, using PADRE sequences, linkers, and adjuvants (heparin-binding hemagglutinin (HBHA)) from *Mycobacterium* sp., β-defensin, ribosomal protein, and flagellin.

### 2.4. Validation of Immune Reaction 

Physicochemical and antigenic/allergenic properties were determined, and a vaccine construct with the best properties was selected for structure modeling using I-TASSER [31]. This structure was validated using Ramachandran plots and docked with HLA-A (PDB ID: 3OX8), HLA-B (PDB ID: 4JQX), and TLR4 receptor (PDB ID: 3FXI) for mapping interactions with immune system. The ClusPro server [32] was employed to assess the construct binding with these receptors. Validation was conducted through immune reaction simulation using C-IMMSIM [33] server. Immune response to the vaccine construct was observed at day 1 and day 30. The simulation spanned 1000 steps, the volume was 10, and the adjuvant was 100 units. The simulation incorporated the vaccine and pathogen protein interaction with human leukocyte antigen (HLA) molecules ‘A0203, A6801, B4402, B4403, DRB1_0101, and DRB1_0411′. The remainder of the parameters were kept at default. 

### 2.5. Cloning

Cloning involves the insertion of the desired DNA sequence into a vector, which serves as a carrier for the genetic material. The designed construct was reverse-translated using JCat (http://www.jcat.de/; accessed 13 May 2023), and the resulting optimized sequence was cloned into pET-28(a)+ vector, using SnapGene software. The pET-28(a)+ vector (available at https://www.snapgene.com/plasmids/pet_and_duet_vectors_(novagen)/pET-28a(%2B); accessed 13 May 2023) is commonly used for in silico protein expression in bacterial systems [34,35] and offers various advantages, such as high-level expression and efficient purification of the target protein.

## 3. Results 

### 3.1. Mimic Prediction

**Figure 1:**
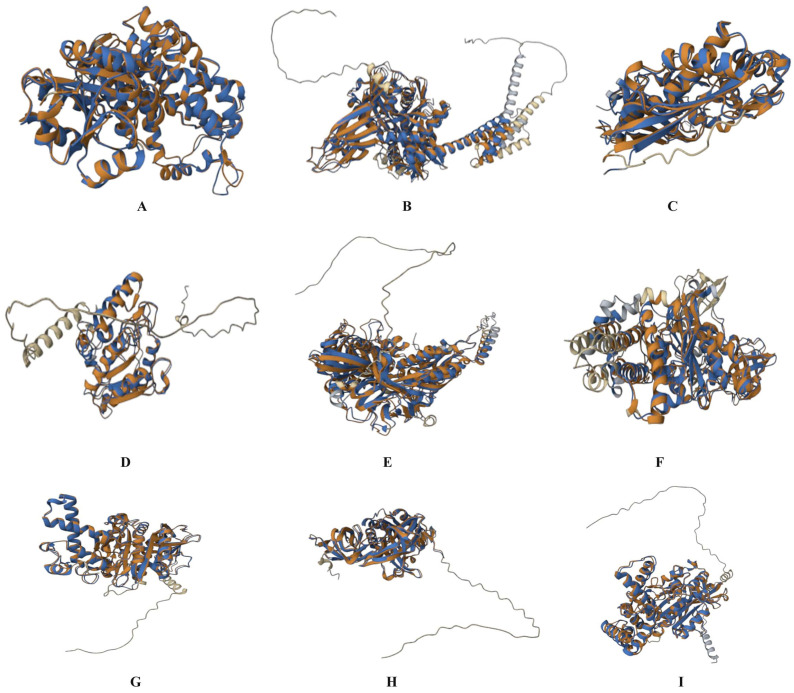
The 3D superposed structures of *S. pneumoniae* homologs (represented in blue) with similar human proteins (represented in brown): (**A**) 6-phosphogluconate dehydrogenase (human: brown, bacteria: blue), (**B**) Chaperone DnaK, (**C**) Methionine adenosyltransferase, (**D**) Uracil-DNA glycosylase, (**E**) GTP-binding protein LepA, (**F**) V-type ATP synthase alpha chain, (**G**) V-type ATP synthase beta chain, (**H**) Translation–elongation factor Tu, and (**I**) ATP synthase F1, alpha subunit.

In total, 13 homologous proteins between *S. pneumoniae* and humans were obtained. A higher number of similar peptides among ATP synthase, chaperone DnaK, and methionine adenosyltransferase implies a greater likelihood of conserved regions and shared functionality (Table 1). Hence, the structures of the proteins were superposed (Figure 1), and the RMSD was obtained. The iPBA server provided slightly lower RMSD values than the TM-align server, possibly due to the difference in their back-end algorithm. The highest RMSD value was observed for the chaperonin GroL protein, while the lowest value was obtained for the ATP synthase F1 beta protein. No direct correlation was found between the RMSD values and the number of similar amino acids, likely due to proteins with a large number of similar folds showing lower RMSDs, even when large sequence similarity was absent.

### 3.2. Pathway Analysis of Homologs

It is known that autoimmune diseases are often triggered by infections, and several protein homologs in our study were associated with both autoimmune and infection pathways (Table 2). Except for Uracil-DNA glycosylase and GTP-binding protein LepA, other proteins were found to exhibit a link with either autoimmune disorders, infections, or both. 6-phosphogluconate dehydrogenase, ATP synthase, and translation–elongation factor Tu were linked with neurodegenerative autoimmune disorders. 6-phosphogluconate dehydrogenase and both chaperones were linked with infections such as HIV and tuberculosis. Protein pathways for studied homologs, linked with both infection and autoimmune disorder, suggest that there is a complex interplay between some infections and the triggering of autoimmunity, which is supported by the literature. 

### 3.3. Mimic-Based Vaccine Design

Similar peptides in human and *S. pneumoniae* homologs, or ‘mimics’, were selected for epitope mapping after determining allergenicity, toxicity, and immunogenicity (Table 3). Uracil-DNA glycosylase and GTP-binding protein LepA mimics were removed from the analysis, as they did not depict any role in autoimmune disorders. For the others, all were determined as non-toxic, but few showed allergenicity. Interleukins have a crucial role in immune response regulation and coordination [52]. Therefore, peptide stimulation of interleukin response was predicted from available servers for IL-4, IL-6, and 13 induction. Peptides 3, 4, 6, 8, and 9 did not induce IL-4 response, while the remainder of the peptides depicted induction. All the peptides displayed IL-6 induction. Except for peptides 6 and 12, the remainder of them depicted IL-13 induction. All these interleukins are allied with autoimmune disorders [53,54,55,56]. 

**Figure 2:**
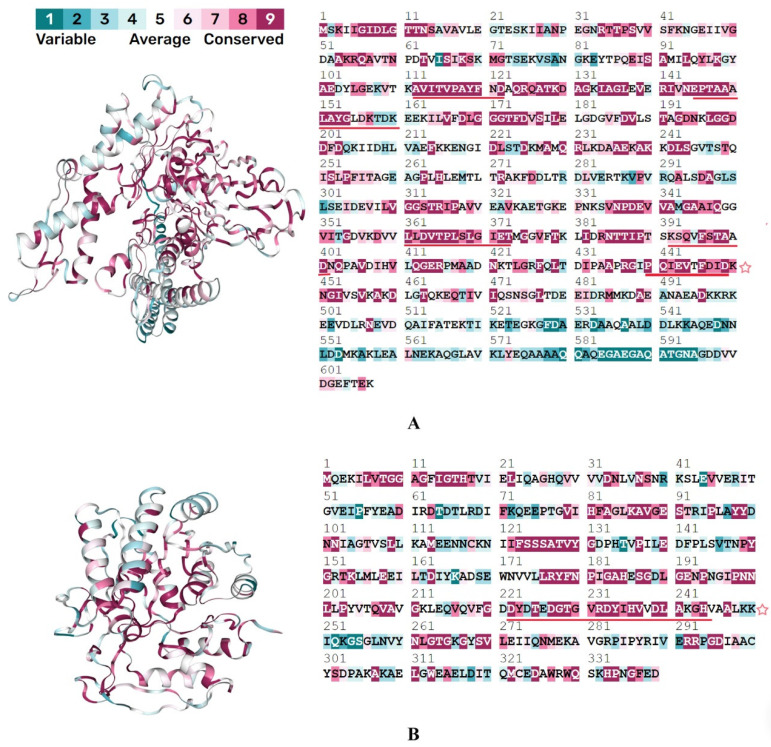
ConSurf-mined evolutionary conservation of (**A**) DnaK and (**B**) UDP-glucose epimerase. Epitopes are underlined in red and those used for vaccine design are depicted by a red star. HLA binding alleles for MHC-I were determined as HLA-A*02:03, HLA-B*44:03, HLA-B*44:02, HLA-A*68:01, HLA-A*02:06, HLA-DQA1*01:01/DQB1*05:01, and HLA-DRB4*01:01 for MHC-II. Eight vaccine constructs were created (Supplementary Table S1), and the one having the highest antigenicity and other desirable properties (C8) was selected for downstream processing.

Mimic-based vaccine construct holds promise for effective immunization and prevention of *S. pneumoniae*-associated diseases. Therefore, two non-allergenic and non-toxic peptides with the highest antigenicity were selected for vaccine design after mapping the evolutionary conservation. Both of them comprised conserved residues (Figure 2). 

### 3.4. C8 Modeling and Docking

**Figure 3:**
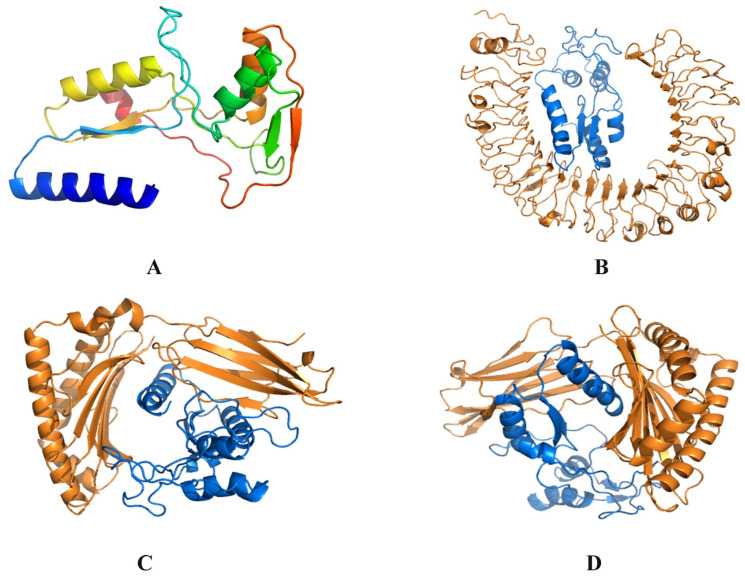
(**A**) The 3D structure of C8, (**B**) C8 docked with TLR4 human receptor, (**C**) C8 docked with HLA-A, and (**D**) C8 docked with HLA-B. All human receptors are depicted by orange and vaccine construct (ligand) is depicted by blue.

The 3D structure of C8 was obtained from I-TASSER. Templates included UDP-glucose 4-epimerase from *Aspergillus nidulans* (PDB ID: 4LIS), *Bacillus anthracis* str. Ames (PDB ID: 2C20), *Brucella abortus* (PDB ID: 4TWR), *Bifidobacterium longum* (PDB ID: 6K0G), *Burkholderia pseudomallei* (PDB ID: 3ENK), *Stenotrophomonas maltophila* (PDB ID: 7KN1), the same enzyme from humans (PDB ID: 1EK5), a mutant of the enzyme from *E.coli* (PDB ID: 1A9Y), galactose epimerase from *Trypanosoma brucei* (PDB ID: 1GY8), and *Saccharomyces cerevisiae* (PDB ID: 1Z45). The top model had a C-score of 0.66, TM-score of 0.80 ± 0.09, and an estimated RMSD value of 3.6 ± 2.5 Å. The model had two sheets, one psi loop, five strands, six helices, one helix–helix interact, nine beta turns, and five gamma turns (Figure 3A). The Ramachandran plot showed that the structure had 77.7% residues in the most favored regions, 18.5% in additionally allowed, and 3.8% in generously allowed regions. No residue was in the disallowed region (Supplementary Figure S1). The RSMD decreased with simulation time, showing stability after 20 nanoseconds of simulation, where it decreased to 3 Å (Supplementary Figure S2A). Average RMSF was also less than 3 Å (Supplementary Figure S2B). Docking with HLA and other receptors showed good binding scores, and the clustering of 1000 poses revealed the best interaction positions with the lowest docking scores. The least score obtained by binding C8 with TLR4 was −1125.7 (Figure 3B), HLA-A was −1340 (Figure 3C), and HLA-B was −1197 (Figure 3D).

### 3.5. Immune Response Simulation

**Figure 4:**
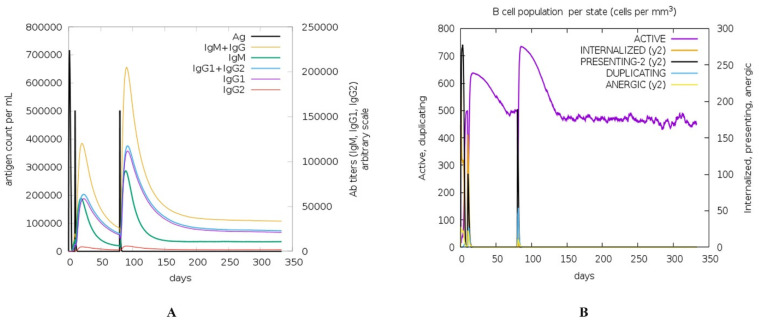
(**A**) Antigen count for the response initiated by C8. (**B**) B-cell population response of C8 after immunization.

The results of C-IMMSIM demonstrated that the vaccine effectively triggered an immune response against multiple epitopes, with a high score on the Parker propensity scale. The top epitopes identified by the simulation were EQIG and STRGRKCCRRKKEAA against allele A0203; EALERAGAKFV against allele A6801; KEAAAKAKF and AEALERAGA against allele B4402; KEAAAKAKF, AEALERAGA, and LERAGAKFV against allele B4403; YCRVRGGRC, FVAAWTLKA, LKAAAGGGS, LERAGAKFV, and FVAAWTLKA against allele DRB1_0101; and FVAAWTLKA against allele DRB1_0411. After 20 days of injection, the IGM + IgG count was 300,000 but doubled after the second injection (50 days). The IgG1 and IgG2 counts reached from 200,000 to near 400,000 (Figure 4A). However, they did not show any alteration upon exposure to pathogen proteins. Active B-lymphocytes showed a similar trend (Figure 4B). This suggests a boost in defense response after the vaccine shot. The results of the simulation are encouraging, and they suggest that the vaccine is likely to be effective in protecting against the pathogen. However, it is important to note that the simulation is just a model, and it is not possible to be sure of the effectiveness of the vaccine until it is tested in clinical trials.

### 3.6. Cloning C8

**Figure 5:**
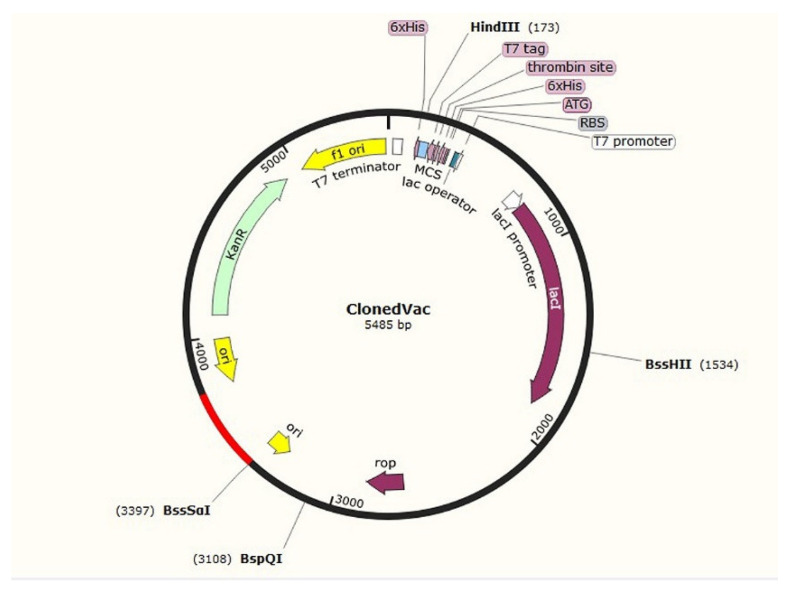
Cloned vaccine construct C8 shown in red in the *E. coli* expression vector.

By employing in silico cloning techniques, researchers can examine factors such as compatibility between the DNA insert and the expression vector, the potential presence of restriction enzyme recognition sites, and predicted secondary structures that may affect the efficiency of cloning. This computational approach saves time and resources by providing insights into the expected outcomes of the cloning process, enabling researchers to optimize their experimental design before proceeding with actual laboratory work. For this purpose, the codon adaptation index of C8 was optimized to 1, resulting in a GC content of 46.54% for this sequence. Restriction enzymes for cloning were BssSɑI and AlwNI. The resulting cloned vaccine construct (C8) was successfully integrated into the *E. coli* expression vector, with a final size of the vector measuring 5485 bp (Figure 5). 

## 4. Discussion

Molecular mimicry plays a substantial role in triggering autoimmune disorders. It occurs when pathogens or foreign agents possess molecular structures that closely resemble self-antigens in the host. This resemblance can potentially disrupt or undermine immune tolerance, leading to a breakdown in the normal regulation of the immune system and the activation of autoreactive immune responses, triggering autoimmune diseases [57]. Previous works suggested that *S. pneumoniae* is involved in autoimmune disorders such as encephalomyelitis [58], rheumatic disorder [59], and Lymphoproliferative syndrome [60]. The lipoteichoic acid and peptidoglycan of this pathogen can be recognized by pattern recognition receptors on immune cells, which triggers the production of pro-inflammatory cytokines and initiates the recruitment of additional immune cells [61,62]. Secondly, virulence factors, such as pneumolysin, can directly damage the host cells and trigger immune responses [63]. It can also induce inflammatory mediators, leading to the activation of adaptive immune responses [61]. The role of some entities has been implicated in triggering autoimmunity, but a comprehensive genome-scale picture is lacking for molecular mimicry in *S. pneumoniae*. We utilized the bioinformatics approach to study this phenomenon, as understanding it can provide valuable insights into the mechanism by which *S. pneumoniae* infection contributes to autoimmune disorders.

We identified 13 homologous proteins between *S. pneumoniae* and humans. Thereafter, we analyzed the potential implications of sequence mimics between them in autoimmune disorders and immune defense activation. Homology analysis revealed several similar peptides among ATP synthase, chaperone DnaK, and methionine adenosyltransferase, indicating the presence of conserved regions and shared functionality. Our findings aligned with previous studies, where heat shock proteins have previously been implicated in molecular mimicry in atherosclerosis [64]. Additionally, their role in molecular mimicry by *C. jejuni* in Guillain–Barré syndrome [65] and *S. typhi* in autoimmunity has been identified [18]. Similarly, molecular chaperone GroEL and V-type ATP synthase have been implicated as molecular mimicry candidates in *Clostridium botulinum* [66]. Another mimic identified in this study, i.e., ATP synthase, has been implicated in autoimmune disorders such as Parkinson’s disease, multiple sclerosis, Alzheimer’s disease, and amyotrophic lateral sclerosis [67]. This suggests that these proteins may play crucial roles in the virulence of several pathogens, as well as in autoimmunity or infection in human systems.

The superposition of protein structures and the calculation of RMSD values provided a snapshot of the structural mimics as well. Pathway analysis revealed associations among the studied homologs, autoimmune disorders, and infections, indicating a complex mechanism between infections and the triggering of autoimmunity. The sequence mimics suggest the possibility of cross-reactivity and immune system activation in the host. In this context, interleukin induction properties of mimic peptides were assessed using available web servers for IL-4, IL-6, and IL-13, as interleukins are involved in autoimmune disorders. IL-4 promotes the differentiation of T-helper 2 and B cells [68] for defense against parasites and is involved in the development of allergic and autoimmune diseases such as Hashimoto’s thyroiditis [69] and psoriasis [70]. Tamgue et al. demonstrated the role of miRNAs in regulating the IL-4 and IL-13 during mycobacterial infection [71]. Chi et al. also reported the role of IL-4 and IL-6 in autoimmune hepatitis [72]. IL-4 and IL-6 have also been implicated in autoimmune thyroid [73]. In *S. pneumoniae,* peptides 3, 4, 6, 8, and 9 did not induce IL-4 response, whereas the remainder of the peptides did. IL-6 has been implicated in an inflammatory response and development of rheumatoid arthritis [74], and all peptide mimics were predicted to induce IL-6 response in this study. This implies that the peptides may have the ability to activate immune cells and promote inflammation, which can further contribute to tissue damage and disease progression, including autoimmune disease. IL-13 plays a role in the pathogenesis of several autoimmune diseases, including rheumatoid arthritis, systemic lupus erythematosus, ulcerative colitis, Sjogren’s syndrome [54], and multiple sclerosis [75]. In total, 11 peptides were predicted to instigate IL-13 production. Hence, these mimic peptides have the potential to contribute to the development or exacerbation of inflammatory processes associated with autoimmune disorders. Additionally, the ability of these mimics to induce multiple interleukin responses, for instance, triggering of IL-6 and IL-13 responses by a single peptide mimic simultaneously, means it can modulate both inflammatory and immune regulatory pathways. This indicates the capacity of mimics to trigger complex immune reactions, disrupt homeostasis, and modulate different aspects of the immune system. This can have broader implications for autoimmune diseases and the development of targeted therapeutic strategies.

Consequently, we utilized antigenic sequence mimics from the dataset for vaccine design, which could potentially be used for immunization and prevention of *S. pneumoniae* infection and autoimmune-related disorders that are triggered by it. Two non-allergenic peptides with high antigenicity were selected for the vaccine design on the basis of evolutionary conservation. The 3D structure of the highest antigenic construct C8 was obtained, and the molecular dynamics simulations confirmed the stability of the structure. Docking with HLA and TLR4 receptors demonstrated good binding scores, and the clustering of poses identified the most favorable interaction positions. TLR4 has been identified as a defense regulator in *S. pneumoniae* infection [76]. It also activates neutrophil extracellular traps that kill *S. pneumoniae*. Thus, binding with it has implications for activating a defense against this bacterium in the human host [77]. HLA genes have been strongly associated with the development of various autoimmune disorders, including celiac disease and type 1 diabetes [78]. Thus, their binding to the sequence mimics of pathogens may also trigger an immune response as well as an autoimmune disorder in the host. This study also provides a thorough understanding of the vaccine characteristics and potential effectiveness based on peptide mimics; however, being a computational study, it has some limitations as well. The algorithms and predictive models used for the analyses depend on the accuracy of the data used to train them, the assumptions made during development and, thus, may have inherent limitations in accurately predicting the molecular interactions and immune responses. Variations in mimicry patterns and immune responses over time may evolve, and computational studies may not capture the full extent of these evolutionary dynamics, limiting the generalizability of the findings. The interactions between hosts and pathogens are also highly complex and dynamic. Computational studies often simplify these interactions by focusing on specific protein sequences or structural motifs, overlooking the intricate interplay of multiple factors involved in immune recognition and response. The varied role of different immune cell types, antigen presentation, and other immune regulatory mechanisms may not be effectively captured by bioinformatics, and, hence, the immune response and the efficacy of the vaccine design may be impacted. Thus, the in silico simulation results are promising, but they are not a substitute for actual experimental validation. We acknowledge this limitation and propose further experimental testing to confirm the findings. Additionally, the sample size of epitopes and HLA alleles evaluated may not fully represent the diversity of potential immune responses. A broader and more diverse sample could provide a more comprehensive understanding of the vaccine construct’s effectiveness.

## 5. Conclusions

Identifying the specific cues for autoimmune disorders through molecular mimicry can aid in elucidating the underlying pathogenic processes and potentially guide the development of targeted therapies for autoimmune conditions. We presented a comprehensive picture of the mimicry mechanism adopted by *S. pneumoniae* through a range of bioinformatics analyses, including epitope mapping, physicochemical property analysis, interleukin stimulation prediction, evolutionary conservation mapping, and docking in this study. We identified 13 homologous proteins between *S. pneumoniae* and humans. Two non-allergenic mimics from two of these proteins, exhibiting high antigenicity, were selected for the vaccine design. The fabricated construct showed stability and the potential to induce immune responses. Our findings should be regarded as preliminary, and their validation through rigorous experimental investigations is essential to ensure their robustness and applicability. We also imply that a broader range of epitopes and HLA alleles would provide a more complete picture. The immune response is highly diverse and influenced by individual genetic variations, including HLA alleles. Therefore, expanding the analysis to encompass a wider range of epitopes and HLA alleles would provide a more representative picture of the potential immune responses and the effectiveness of the vaccine construct in a broader population. This would enhance the generalizability and applicability of the findings. We also propose similar studies on other pathogens.

## Figures and Tables

**Table 1:** Statistics for sequence and structure similarity between human and *S. pneumoniae* homologs.

Serial No.	Name	UniProt ID of Human Homolog	NCBI Accession of Bacterial Homolog	Bacterial Protein Structure AlphaFold ID	No. of Similar Peptides (Length ≥ 10)	Molecular Mimic Region (Length ≥ 10)	Superposed Protein RMSD (iPBA)	TM-Align RMSD
1	6-phosphogluconate dehydrogenase	P52209.3	ARD34052.1	J1NT41	3	LIQAQRDYFGAHTY SKIISYAQGF VKMVHNGIEYGDMQLI	0.79	0.91
2	Chaperone protein DnaK	P38646	ARD34182.1	C1CQ18	5	PQIEVTFDID KSQVFSTAAD LLDVTPLSLGIET EPTAAALAYGLDK AVITVPAYFND	1.36	2.6
3	Methionine adenosyltransferase	Q00266	ARD34414.1	B2INE5	4	GAGDQGLMFG GRFVIGGPQGD TGRKIIVDTYGG HGGGAFSGKD	0.86	1.24
4	Uracil-DNA glycosylase	P13051	ARD34802.1	C1CRP5	2	VKVVILGQDPYHGP AHPSPLSVYRGF	1.35	1.73
5	GTP-binding protein LepA	Q8N442	ARD34823.1	B1IC02	2	DHGKSTLADR LIDTPGHVDF LVKMDILLNG	2.13	2.76
6	V-type ATP synthase alpha chain	P38606	ARD34956.1	B1ICT1	0	-	1.53	3.12
7	V-type ATP synthase beta chain	P21281	ARD34955.1	B1ICC8	3	EALREVSAAR DDITHPIPDLTGYITEGQI PPINVLPSLSRL	0.84	1.04
8	Translation elongation factor Tu	P49411	ARD35089.1	C1CSB0	3	GTIGHVDHGKTTLTAAIT PGHADYVKNMITG DGPMPQTREH	0.81	0.81
9	ATP synthase F1, alpha subunit	P25705	ARD35106.1	B1ICT1	6	LVPIGRGQRELIIGDRQTGKT YDDLSKQAVAYR SLLLRRPPGREA PGDVFYLHSRLLER TNVISITDGQIFL FGSDLDAATQ	0.74	1.04
10	ATP synthase F1, beta subunit	P06576	ARD35104.1	B8ZLA9	7	GLFGGAGVGKTVLI SVFAGVGERTREGNDLY GQMNEPPGAR EGQDVLLFIDNIFRFTQAGSEVSALLGR PSAVGYQPTLAT LGIYPAVDPL LQDIIAILGMDELS	0.72	0.88
11	UDP-glucose 4-epimerase	Q14376	ARD35197.1	A5MBZ8	2	VIHFAGLKAVGES DYDTEDGTGVRDYIHVVDLAKGH	0.84	0.99
12	2,3-bisphosphoglycerate-dependent phosphoglycerate mutase	P15259	ARD35240.1	C1C8P5	1	WRLNERHYGGLTG	1.03	1.03
13	chaperonin GroL	P10809	ARD35488.1	J1NT41	2	AGDGTTTATVL DALNATRAAVEEGIV	1.81	3.81

**Table 2:** Database and literature information for *S. pneumoniae* and human-homologous-protein-linked pathways involved in autoimmune disorders.

Serial No.	Protein Homolog	PHAROS		PATHDIP		Literature	
		Autoimmunity Pathway	Infection Pathway	Autoimmune Pathway	Infection Pathway	Autoimmune Pathway	Infection Pathway
1	6-phosphogluconate dehydrogenase	Diabetes mellitus	-	Huntington’s disease, Rheumatoid arthritis, Parkinson’s disease, Alzheimer’s disease, Non-alcoholic steatohepatitis	Vibrio infection, HIV, Influenz, Tuberculosis, Human papillomavirus infection	Nephritogenic autoimmunity [36]	-
2	Chaperone protein DnaK	Autoimmune disease, Parkinson’s disease	Perinatal necrotizing enterocolitis, HIV, Tuberculosis	-	-	Guillain–Barré syndrome, Multiple sclerosis, Systemic lupus erythematosus [37,38]	-
3	Methionine adenosyltransferase	Type 2 diabetes mellitus, demyelinating diseases, MODY, Psoriasis, Fatty liver, or Non-alcoholic steatohepatitis	-	-	-	-	Herpes simplex type 1 [39], Poxvirus [40]
4	Uracil-DNA glycosylase	-	-	-	-	-	-
5	GTP-binding protein LepA	-	-	-	-	-	-
6	V-type ATP synthase alpha chain	Psoriasis	-	Alzheimer, Parkinson’s disease, Huntington’s disease, Rheumatoid arthritis	HPV, *Vibrio cholerae*	-	Rabies [41]
7	V-type ATP synthase beta chain	IgA glomerulonephritis	-	Huntington’s disease, Rheumatoid arthritis	HPV	-	-
8	Translation–elongation factor Tu	-	-	Huntington’s disease, Parkinson’s disease	HCV, HBV, Legionellosis, *E. coli*, *V. cholerae*	Sjogren’s syndrome [42], Crohn’s disease [43]	*Streptococcus pneumoniae* [44], Other microbes pathogenesis [45]
9	ATP synthase F1, alpha subunit	Alzheimer’s disease	-	-	-	Sjogren’s syndrome, Crohn’s disease [43]	-
10	ATP synthase F1, beta subunit	-	-	Alzheimer’s disease, Huntington’s disease, Parkinson’s disease, Non-alcoholic fatty acid liver diseases	Epstein–Barr virus infection, HBV, HCV, HPV, Measles, Legionellosis, *E. Coli*	Autoimmune myocarditis [46]	-
11	UDP-glucose 4-epimerase	Psoriasis, interstitial cystitis	Tinea corporis, Tinea pedis	-	-	-	*Haemophilus influenzae* [47]
12	2,3-bisphosphoglycerate-dependent phosphoglycerate mutase	Juvenile dermatomyositis	-	-	-	-	Papillomavirus infection [48]
13	chaperonin GroL	Allergic rhinitis	Tuberculosis, HIV	-	-	Type-1 diabetes, Juvenile chronic arthritis, Atherosclerosis, Crohn disease, Rheumatoid arthritis, Systemic lupus erythematosus, Sjogren’s syndrome, Hashimoto thyroiditis, and Myasthenia gravis [49], Autism [50]	*P. aeruginosa* and *S. aureus* [51]

**Table 3:** Physicochemical and other property analysis of shortlisted peptides.

Serial No.	Antigenic Score	Sequence	Length	Immunogenicity Score	SVM Score for Toxicity	Hydrophobicity	Hydropathicity	Hydrophilicity	Charge	Mol wt	Allergenicity
1	1.5685	**PQIEVTFDID**	10	0.43	−1.33	−0.04	−0.03	0.12	−3.00	1176.43	Non-allergen
2	0.8794	LLDVTPLSLGIET	13	0.10	−1.30	0.12	0.98	−0.38	−2.00	1370.81	Allergen
3	0.5923	GAGDQGLMFG	10	−0.14	−1.07	0.09	0.17	−0.29	−1.00	952.20	Allergen
4	1.2285	HGGGAFSGKD	10	−0.06	−1.31	−0.10	−0.84	0.28	0.50	932.10	Allergen
5	0.5672	GTIGHVDHGKTTLTAAIT	18	0.28	−1.54	−0.00	0.12	−0.27	1.00	1793.29	Allergen
6	0.5607	DGPMPQTREH	10	−0.17	−0.50	−0.41	−2.06	0.70	−0.50	1167.40	Allergen
7	0.7650	SLLLRRPPGREA	12	0.20	−0.59	−0.36	−0.68	0.53	2.00	1364.78	Allergen
8	1.0099	FGSDLDAATQ	10	0.07	−1.42	−0.08	−0.22	0.08	−2.00	1024.18	Non-allergen
9	1.0376	PSAVGYQPTLAT	12	0.01	−1.47	0.03	0.08	−0.58	0.00	1204.51	Allergen
10	0.5485	LGIYPAVDPL	10	0.12	−1.22	0.19	0.97	−0.67	−1.00	1057.40	Non-allergen
11	0.9488	**DYDTEDGTGVRDYIHVVDLAKGH**	23	0.50	−0.62	−0.20	−0.80	0.39	−3.00	2576.07	Non-allergen
12	0.7924	WRLNERHYGGLTG	13	0.24	−1.09	−0.26	−1.21	−0.08	1.50	1558.92	Allergen
13	1.9333	AGDGTTTATVL	11	0.25	−0.75	0.04	0.41	−0.26	−1.00	1006.23	Allergen

## Data Availability

All the data used or generated in this study are provided as accession numbers or relevant information as tables in the manuscript.

## Supplementary Materials

The following supporting information can be downloaded at: https://www.mdpi.com/article/10.3390/pathogens12070857/s1, Table S1: Vaccine constructs made by selected mimics; Figure S1: Ramachandran plot of C8; Figure S2: (A) RMSD plot of C8, after 100 ns of simulation. Each nanosecond corresponds to 10 frames. (B) RMSF plot of C8, after 100 ns of simulation.
