# Supplementary material for: Molecular Mimicry Mapping in Streptococcus pneumoniae: Cues for Autoimmune Disorders and Implications for Immune Defense Activation
Source: Pathogens. 2023 Jun 21;12(7):857. doi: 10.3390/pathogens12070857 (PMC10383125; doi:10.3390/pathogens12070857)
Supplement: Supplementary file 1 [file pathogens-12-00857-s001.zip › pathogens-2436793-supplementary.pdf]

Supplementary Table S1. Vaccine constructs made by selected mimics.

Serial no.	Construct	Length	allergenicity	antigenic	stability	GRAVY	Molecular weight
1	>C1 EAAAKMAENSNIDDIKAPLLAALGAADLALATVNELITNLRERAEETRRSRVEESRARLTKLQEDLPEQLTELREKFTAEELRKAAEGYLEAATSELVERGEAALERLRSQQSFEEVSARAEGYVDQAVELTQEALGTVASQVEGRAAKLVGIELEAAAKAKFVAAWTLKAAAGGGS**PQIEVTFDID**GGGSAKFVAAWTLKAAAGGGSDYDTEDGTGVRDYIHVVDLAKGHGGGS**HEYGAEALERAG**AKFVAAWTLKAAAGGGS	264	Non-allergen	0.96	42.62 (unstable)	-0.27	27731.81
2	>C2 EAAAKMAKLSTDELLDAFKEMTLLELSDFVKKFEETFEVTAAAPVAVAAAGAAPAGAAVEAAEEQSEFDVILEAAGDKKIGVIKVVREIVSGLGLKEAKDLVDGAPKPLLEKVAKEAADEAKAKLEAAGATVTVKEAAAKAKFVAAWTLKAAAGGGSDYDTEDGTGVRDYIHVVDLAKGHGGGS**HEYGAEALERAG**PQIEVTFDIDGGGS**HEYGAEALERAG**AKFVAAWTLKAAAGGGS	239	Allergen	0.86	20.83 (stable)	-0.02	24358.38
3	>C3 EAAAKMAENPNIDDLPAPLLAALGAADLALATVNDLIANLRERAEETRAETRTRVEERRARLTKFQEDLPEQFIELRDKFTTEELRKAAEGYLEAATNRYNELVERGEAALQRLRSQTAFEDASARAEGYVDQAVELTQEALGTVASQTRAVGERAAKLVGIELEAAAKAKFVAAWTLKAAAGGGSDYDTEDGTGVRDYIHVVDLAKGHGGGSAKFVAAWTLKAAAGGGSPQIEVTFDIDGGGS**HEYGAEALERAG**AKFVAAWTLKAAAGGGS	273	Non-allergen	0.95	36.68 (stable)	-0.315	28850.03

4	>C4 EAAAKMAENSNIDDIKAPLLAALGAADLALATVNELITNLRERAEETRRSRVEESRARLTKLQEDLPEQLTELREKFTAEELRKAAEGYLEAATSELVERGEAALERLRSQQSFEEVSARAEGYVDQAVELTQEALGTVASQVEGRAAKLVGIELEAAAKAKFVAAWTLKAAAGGGSAKFVAAWTLKAAAGGGSDYDTEDGTGVRDYIHVVDLAKGHGGGS**HEYGAEALERAG**PQIEVTFDIDGGGS**HEYGAEALERAG**AKFVAAWTLKAAAGGGS	276	Non-allergen	0.97	39.00 (stable)	-0.30	29016.16
5	>C5 EAAAK**MAQVINTNSLSLLTQNNLNKSQSSLSSAIERLSSGLRINSAKDDAAGQAIANRFTSNIKGLTQASRNANDGISIAQTTEGALNEINNNLQRVRELSVQATNGTNSDSDLKSIQDEIQQRLEEIDRVSNQTQFNGVKVLSQDNQMKIQVGANDGETITIDLQKIDVKSLGLDGFNV**EAAAKAKFVAAWTLKAAAGGGSDYDTEDGTGVRDYIHVVDLAKGHGGGSAKFVAAWTLKAAAGGGSPQIEVTFDIDGGGS**HEYGAEALERAG**AKFVAAWTLKAAAGGGS	289	Non-allergen	1.04	27.29 (stable)	-0.35	30125.21
6	>C6 EAAAKMAENPNIDDLPAPLLAALGAADLALATVNDLIANLRERAEETRAETRTRVEERRARLTKFQEDLPEQFIELRDKFTTEELRKAAEGYLEAATNRYNELVERGEAALQRLRSQTAFEDASARAEGYVDQAVELTQEALGTVASQTRAVGERAAKLVGIELEAAAKAKFVAAWTLKAAAGGGSPQIEVTFDIDGGGSAKFVAAWTLKAAAGGGSDYDTEDGTGVRDYIHVVDLAKGHGGGS**HEYGAEALERAG**AKFVAAWTLKAAAGGGS	273	Non-allergen	0.95	36.68 (stable)	-0.32	28850.03
7	>C7 EAAAKMAENSNIDDIKAPLLAALGAADLALATVNELITNLRERAEETRRSRVEESRARLTKLQEDLPEQLTELREKFTAEELRKAAEGYLEAATSELVERGEAALERLRSQQSFEEVSARAEGYVDQAVELTQEALGTVASQVEGRAAKLVGIELEAAAKAKFVAAWTLKAAAGGGSDYDTEDGTGVRDYIHVVDLAKGHGGGS**HEYGAEALERAG**PQIEVTFDIDGG	259	Non-allergen	0.93	40.86 (unstable)	-0.35	27428.32

	GSHEYGAEALERAGAKFVAAWTLKAAAGGGS						
8	>C8 EAAAKGIINTLQKYYCRVRGGRCAVLSCLPKEEQIGKCSTRGRKCCRRKKEAAAKAKFVAAWTLKAAAGGGSDYDTEDGTGVRDYIHVVDLAKGHGGGSAKFVAAWTLKAAAGGGSPQIEVTFDIDGGGSHEYGAEALERAGAKFVAAWTLKAAAGGGS	159	Non-allergen	1.25	31.69 (stable)	-0.24	16382.58

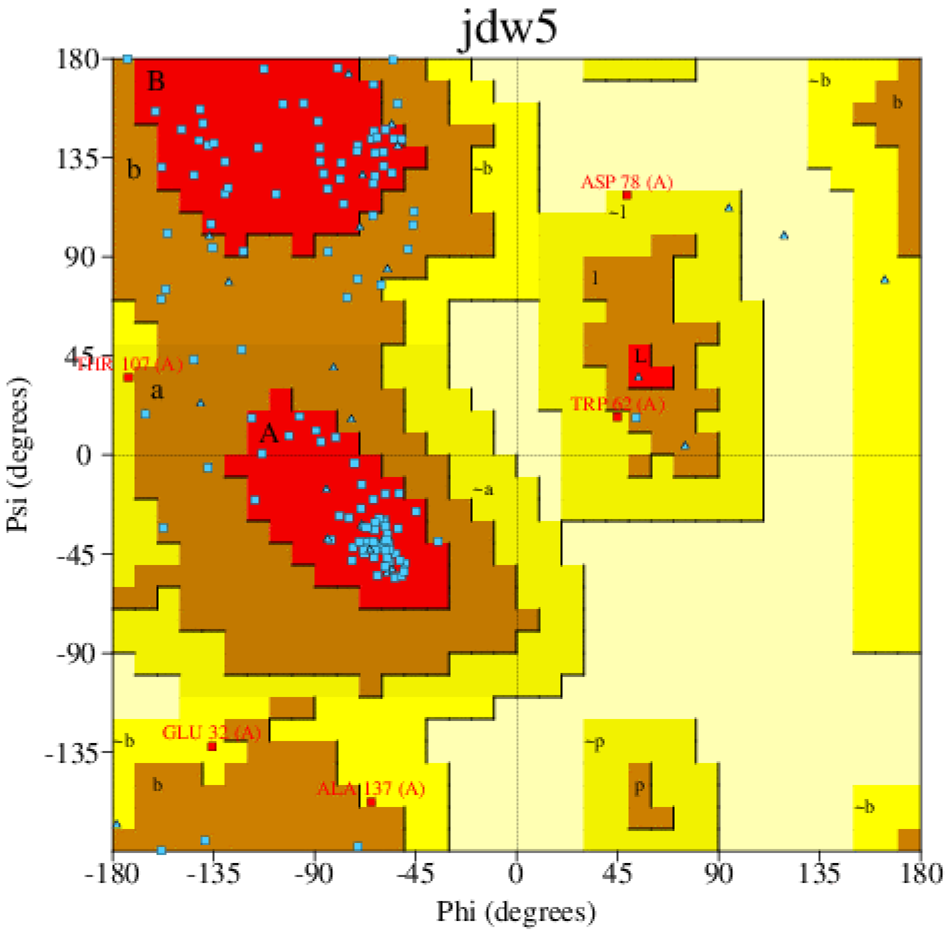

Supplementary Figure S1. Ramachandran plot of C8.

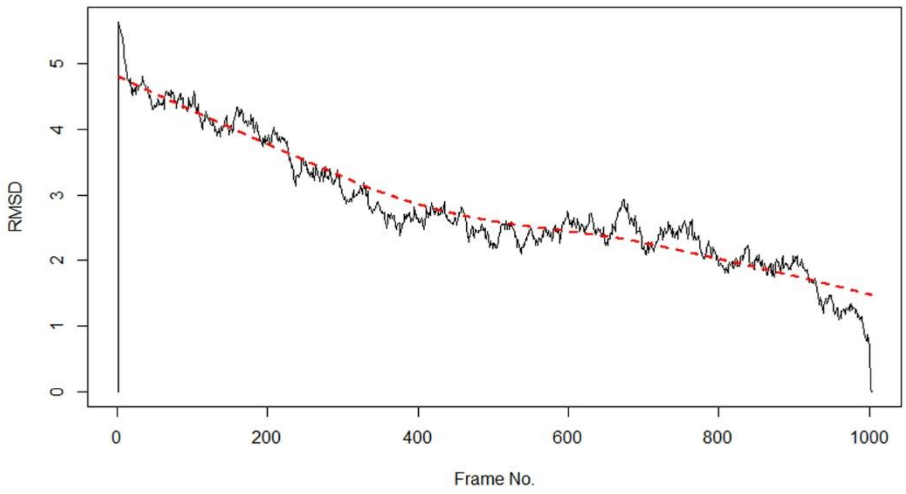

A

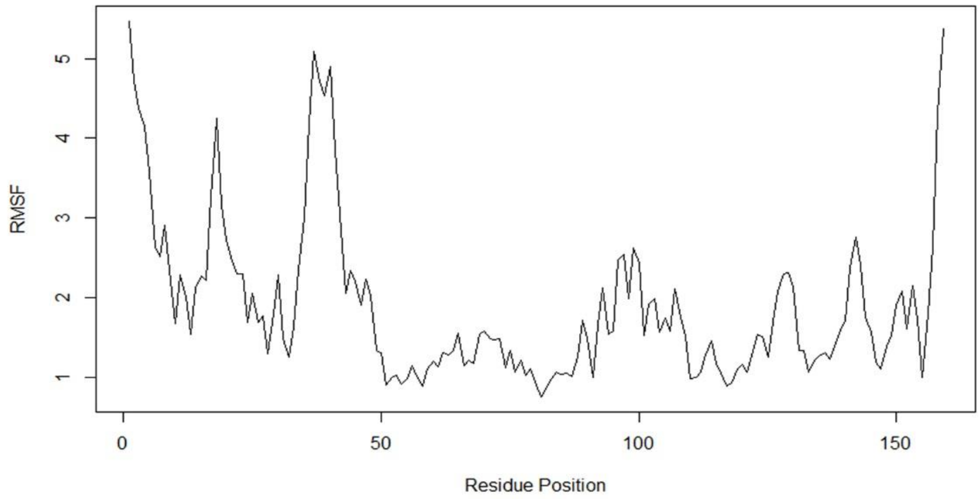

B

Supplementary Figure S2. (A) RMSD plot of C8, after 100 ns of simulation. Each nanosecond corresponds to 10 frames. (B) RMSF plot of C8, after 100 ns of simulation.
